# Editorial: Non-biomedical perspectives on pain and its prevention and management – volume II

**DOI:** 10.3389/fpain.2026.1924013

**Published:** 2026-08-04

**Authors:** Mark I. Johnson, Kate Thompson, Antonio Bonacaro, Emmanouil Georgiadis

**Affiliations:** 1 Centre for Pain Research, School of Health, Leeds Beckett University, Leeds, United Kingdom; 2 Department of Medicine and Surgery, School of Nursing, University of Parma, Parma, Italy; 3 School of Social Sciences and Humanities, University of Suffolk, Ipswich, United Kingdom

**Keywords:** care environments, lived experience, non-biomedical perspectives, pain, pain ontology, relational care, social determinants of health

## Introduction

Pain remains a major public health challenge worldwide, contributing substantially to disability, reduced quality of life, and social and economic burden. While biomedical approaches have advanced understanding of nociception and pain-related pathology, there is increasing recognition that such perspectives alone are insufficient to account for the persistence, variability, and inequitable distribution of pain. Social, cultural, relational, and environmental factors play a central role in shaping how pain is experienced, interpreted, prevented, and managed. This Research Topic, *Non-biomedical perspectives on pain and its prevention and management – Volume II*, builds on this recognition by bringing together a diverse collection of contributions that explore pain beyond narrowly biomedical framings.

Volume I of this Research Topic demonstrated the breadth and legitimacy of non-biomedical approaches to pain, highlighting perspectives drawn from social sciences, humanities, systems thinking, and community-based practice. Volume II extends this work by further examining how such perspectives can inform prevention and management, particularly through attention to upstream conditions, relational processes, and care environments. Rather than proposing a single alternative model of pain, this Research Topic reflects the plurality of non-biomedical perspectives and their relevance to real-world pain contexts.

In methodological terms, the contributions to this Research Topic largely take an exploratory and interpretive approach, focusing on developing ideas and understanding experience rather than on testing specific interventions or outcomes. Many papers examine how pain is recognised, understood, and lived with in situations where standard biomedical measures on their own do not fully capture what matters to people. Others use analytic or evaluative approaches to place pain within wider social, relational, and systemic settings. Although a small number of studies focus on interventions or evaluations, these are situated within a broader non-biomedical perspective that emphasises care environments, communication, and lived experience. Overall, the Research Topic reflects a field that is concerned with learning, reflection, and opening up new ways of thinking about pain, rather than with providing final answers through testing alone. Future research can build on this work by bringing together experience-focused approaches with longer term, comparative, or mixed methods designs, and by paying greater attention to how non-biomedical perspectives are taken up, supported, or limited within different services, settings, and policy contexts, helping to connect new ways of understanding pain with changes in practice.

## Overview of contributions

Across the contributions, several overlapping themes can be identified. These themes do not represent discrete categories but rather points of emphasis that reflect different ways of relocating where pain is recognised, understood, and addressed.

### Making pain visible: recognition, meaning, and expression

Several contributions focus on how pain is recognised, interpreted, and made visible, particularly in contexts where conventional assessment tools or biomedical indicators may be limited. These papers highlight that pain is not always self-evident and that its recognition often depends on interpretive, communicative, and cultural processes.

Pain in later life and in cognitive impairment is addressed by Kruijer et al. in *Observing and treating pain in people living with dementia in long-term care facilities*, which draws attention to the challenges of identifying and responding to pain when verbal self-report is constrained. This work highlights the importance of observational, relational, and contextual approaches to pain recognition in care settings.

The meaning attributed to pain is further explored by Wong et al. in *The meaning of manageable neuropathic pain after SCI*, which examines how individuals living with spinal cord injury understand and live with pain that is not eliminated but rendered manageable. This contribution highlights that pain outcomes cannot be reduced to intensity alone and that meaning plays a key role in how pain is experienced and integrated into daily life.

Complementing these perspectives, three papers explore expressive and interpretive modalities that extend beyond conventional clinical language. *Putting pain into words: The Psalms of Lament as an aid for the alexithymic* by Bäckryd examines the role of metaphor, narrative, and shared cultural texts in articulating pain that is otherwise difficult to express. *Exploring Pain and Suffering Through Spatial Acousmatic Music* by Stavropoulos and Johnson considers how artistic and auditory experiences can offer alternative ways of engaging with pain and suffering. *Exploring magical thinking in the context of pain*, by Heckemann et al. examines symbolic and imaginative meaning-making often dismissed or pathologised in clinical discourse. Through reflective dialogue between a nurse researcher, a writer-practitioner, and a pain scientist the authors explore magical thinking not as belief in altering physical reality, but as a personal orientation that supports sense-making, emotional regulation, and relationships with self, others, and the wider world. By situating magical thinking alongside recognised psychological and cultural practices, this contribution invites broader reflection on the role of symbolism, ritual, and imagination in living with chronic pain.

Taken together, these contributions highlight that making pain visible often requires modes of expression and interpretation that extend beyond conventional biomedical or clinical language.

### Pain as relational and socially mediated

A second group of papers emphasises the relational and social dimensions of pain, aligning with the Research Topic's focus on moving beyond individualised accounts of pain experience and responsibility.

The value of collaboration is explored by Andréll et al. in *The importance of partnership in chronic pain – co-creation of tomorrow's pain care*, which highlights co-creation across research, implementation, and knowledge dissemination. This perspective situates pain care as a shared endeavour involving patients, clinicians, researchers, and communities.

Relational processes are further examined by Świdrak in *Healing synchrony? Potential benefits of interpersonal synchrony for chronic pain management*, which explores how interpersonal alignment and coordination may influence pain experience and management. This work draws attention to subtle, often overlooked social dynamics that shape pain encounters.

Together with the contribution on pain in dementia care by Kruijer et al., these papers emphasise that pain recognition, interpretation, and management are inherently relational processes. They challenge models that locate pain solely within individual bodies and instead highlight the social contexts through which pain is understood and addressed.

### Pain patterned by place, population, and context

Several contributions examine how pain is patterned across populations and contexts, reflecting the Research Topic's emphasis on upstream determinants and social ecology.

*Exploring Chronic Pain: Demographic and Social Determinants of Health Insights in Maine* by Ou et al. provides population-level insights into how social and demographic factors shape chronic pain prevalence and experience. This work highlights the uneven distribution of pain and the importance of contextual factors such as geography, socioeconomic status, and access to resources.

Heterogeneity within diagnostic categories is addressed by Åkerblom et al. in *Identifying and characterizing clinical subgroups in individuals with endometriosis*, which demonstrates the diversity of pain experiences within a single medical condition. Similarly, Bretonneau et al. explores intervention effects within a specific physiological and social context in *Whole-body cryostimulation exposures effectively alleviate menstrual-related pain and associated sleep disturbances in young women*.

A second contribution by Åkerblom et al. *Long-term pain and health economic outcomes in adults receiving multidisciplinary CBT for chronic pain* examines the long-term interplay between psychological processes, pain outcomes, and economic factors. Together, these contributions emphasise that pain cannot be understood independently of population-level patterning, contextual influences, and social conditions.

### Care design and the shaping of pain outcomes

A further set of papers focuses on how care practices, information, and systems are designed, addressing the Research Topic's concern with prevention and management rather than treatment alone.

Information provision is examined by Stamenkovic et al. in *The information that patients, their families, and medical staff wish to know about postoperative pain and its management*, highlighting mismatches between informational needs and current practices. Educational and participatory approaches to care are explored by Ayres et al. in *The Knee-SCHOOL: A brief patient-centered multidisciplinary educational program for knee osteoarthritis*, which emphasises patient-centred learning and shared understanding.

The integration of broader sources of meaning into pain care is addressed by Perrin et al. in *Developing a best practice guide for integrating spiritual care interventions in chronic pain therapy*. This contribution highlights how attention to spiritual and existential dimensions may complement other approaches to pain management.

Collectively, these papers draw attention to care environments as active influences on pain outcomes, suggesting that prevention and management depend not only on interventions but also on how care is organised, communicated, and experienced.

### Reflecting on non-biomedical perspectives

**Figure 1:**
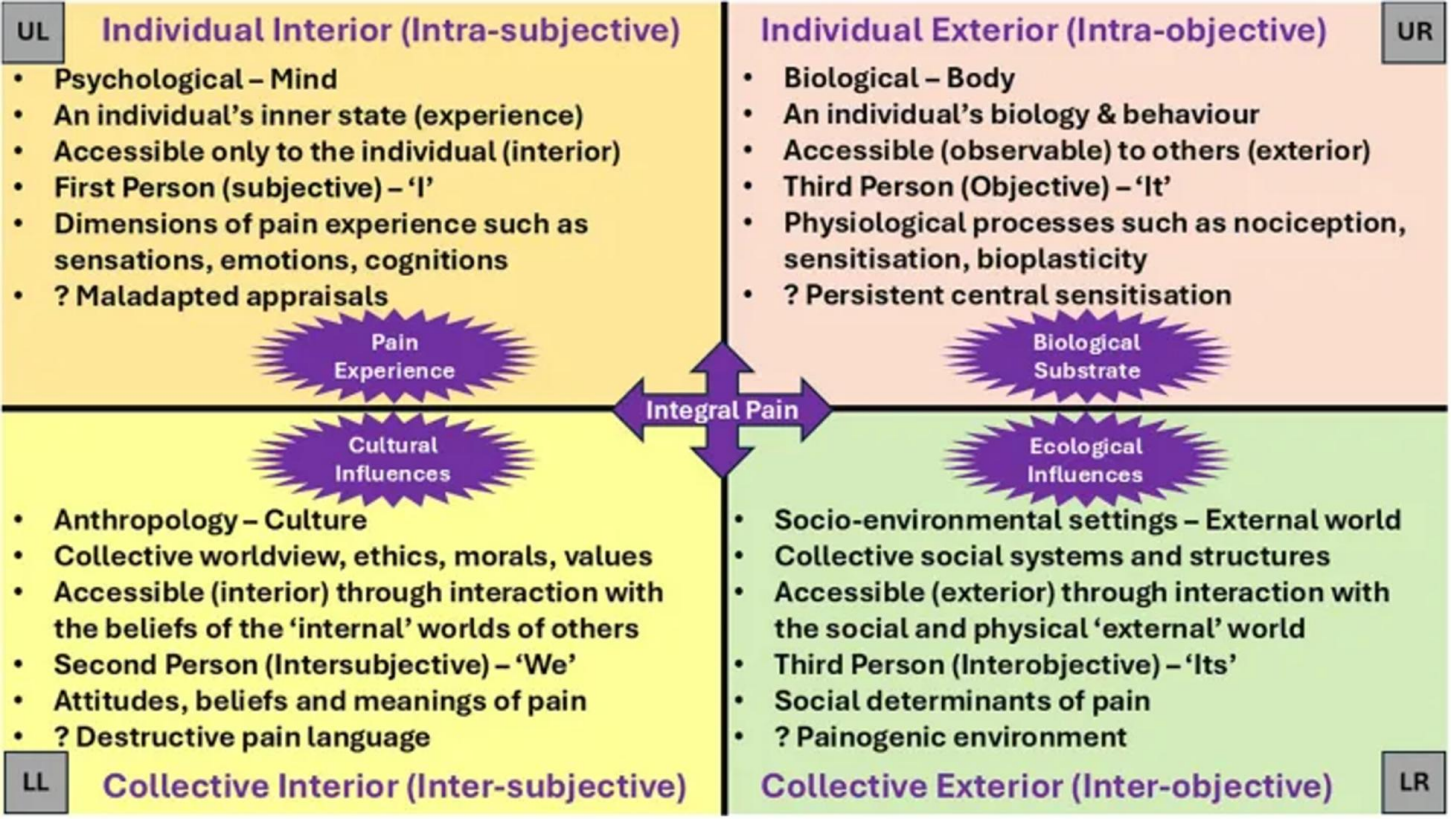
Taken from An integral vision of pain and Its persistence by Johnson. The quadrants framework offers a comprehensive lens through which to understand the multifaceted nature of pain. The Upper Right (UR) quadrant represents the objective, observable aspects of the individual, including neuroscientific and biological mechanisms that govern bodily functions and behaviours. In contrast, the Upper Left (UL) quadrant represents the subjective, psychological, and experiential aspects of the individual's inner life. The Lower Left (LL) quadrant encompasses the intersubjective perspective, highlighting the cultural, moral, and relational aspects that shape meaning within collective society. Finally, the Lower Right (LR) quadrant captures the interobjective perspective, focusing on systemic, structural, and environmental aspects of the broader socio-ecological context. Together, these quadrants provide a holistic framework for understanding pain as a complex interplay of individual and collective, internal and external factors.

One contribution explicitly reflects on the conceptual breadth of non-biomedical approaches. *An Integral Vision of Pain and Its Persistence* by Johnson offers a whole-person, whole-system perspective that invites readers to consider how biological, psychological, social, and contextual factors interact over time. Positioned alongside the other contributions, this paper provides a reflective lens rather than a unifying framework, encouraging dialogue across perspectives rather than seeking conceptual closure (Figure 1).

## Potential impact

Together, the contributions in this Research Topic reflect the growing breadth and maturity of non-biomedical pain research. Rather than advancing a single alternative account of pain, Volume II highlights multiple ways in which attention to meaning, relationships, populations, and systems can enrich understanding of pain prevention and management. These papers collectively invite continued exploration of how pain is shaped by social, cultural, and environmental conditions, and how care practices might respond to these realities.

As the field continues to evolve, non-biomedical perspectives will remain essential for addressing the persistence and inequities of pain. This Research Topic offers an orientation toward that broader landscape, inviting readers to engage with the diverse contributions and consider their implications for future research, practice, and policy.

